# Psychoeducational Social Anxiety Mobile Apps: Systematic Search in App Stores, Content Analysis, and Evaluation

**DOI:** 10.2196/26603

**Published:** 2021-09-21

**Authors:** Trent Ernest Hammond, Lisa Lampe, Andrew Campbell, Steve Perisic, Vlasios Brakoulias

**Affiliations:** 1 Nepean Clinical School Faculty of Medicine and Health The University of Sydney Kingswood, New South Wales Australia; 2 School of Medicine and Public Health College of Health, Medicine and Wellbeing University of Newcastle Callaghan, New South Wales Australia; 3 Cyberpsychology Research Group, Biomedical Informatics and Digital Health School of Medical Sciences, Faculty of Medicine and Health The University of Sydney Camperdown, New South Wales Australia; 4 Mental Health Outreach Centre South Western Sydney Local Health District Campbelltown, New South Wales Australia; 5 School of Medicine Western Sydney University Blacktown, New South Wales Australia

**Keywords:** anxiety, app, cell phone, mobile app, mobile phone, SAD, smartphone, social anxiety, social phobia, tablet

## Abstract

**Background:**

The wide use of mobile health apps has created new possibilities in social anxiety education and treatment. However, the content and quality of social anxiety apps have been quite unclear, which makes it difficult for people to choose appropriate apps to use on smartphones and tablets.

**Objective:**

This study aims to identify the psychoeducational social anxiety apps in the two most popular Australian app stores, report the descriptive and technical information provided in apps exclusively for social anxiety, evaluate app quality, and identify whether any apps would be appropriate for people with social anxiety or others who know someone with social anxiety.

**Methods:**

This systematic stepwise app search was guided by the PRISMA (Preferred Reporting Items for Systematic Reviews and Meta-Analyses) standards and entailed searching for, identifying, and selecting apps in the Australian Apple App and Google Play Stores; downloading, using, and reviewing the identified apps; reporting technical and descriptive information in the app stores, an online app warehouse, and individual apps; evaluating app quality; and deciding whether to recommend the use of the apps.

**Results:**

In the app stores, 1043 apps were identified that contained the keywords *social anxiety*, *social phobia*, or *shyness* in their names or descriptions. Of these, 1.15% (12/1043) were evaluated (3 iOS apps and 9 Android apps). At the time of evaluation, the apps were compatible with smartphones and tablet devices; 9 were free to download from the app stores, whereas 3 were priced between US $2.95 (Aus $3.99) and US $3.69 (Aus $5.00). Among the evaluated apps, 3 were intended for treatment purposes, 3 provided supportive resources, 1 was intended for self-assessment, and the remaining 5 were designed for multiple purposes. At the time of downloading, app store ratings were available for 5 apps. The overall app quality was acceptable according to the Mobile App Rating Scale (MARS). On the basis of the MARS *app quality rating* subscale (sections A-D), the apps functioned well in performance, ease of use, navigation, and gestural design. However, app quality was less favorable when rated using the MARS *app subjective quality* subscale (section E).

**Conclusions:**

The psychoeducational social anxiety apps evaluated in our study may benefit people with social anxiety, health professionals, and other community members. However, given that none of the apps appeared to contain empirical information or were shown to clinically reduce social anxiety (or aid in managing social anxiety), we cannot recommend their use. App accessibility could be improved by developing apps that are free and available for a wider range of operating systems, both between and within countries and regions. Information communication and technology professionals should collaborate with academics, mental health clinicians, and end users (ie, co-design) to develop current, evidence-based apps.

## Introduction

### Background

Social anxiety disorder (SAD) is characterized by the avoidance of social interactions that involve perceived scrutiny by others and potential embarrassment [1]. Signs and symptoms of SAD include avoidance of social activities because of anxiety, blushing, problems in making conversation, being unable to think of anything to say, reduced eye contact, nausea, rapid heartbeat, sweating, and dizziness [2]. People with SAD tend to experience problems in their daily activities [3], have poorer educational outcomes [4,5], are less productive at work, and subsequently have decreased employment prospects [1]. Surveys have reported that between 82.2% (n unavailable; total survey N=43,093) [6] and 88% (73/83) [7] of people with SAD had a diagnosis of at least one other mental disorder during the 12-month period before completing the surveys.

National surveys in the United States and Australia (countries with similar sex ratios and age structures) [8] suggest that SAD [9] affects tens of millions of people worldwide [10,11]. The US National Comorbidity Survey Replication [10] and Australian National Survey of Mental Health and Wellbeing (NSMHWB) [11] reported that SAD is common, with 12-month prevalence rates estimated at 6.80% (631/9282) for people aged 18 years and over and 4.70% (752,719/16,015,300) for people aged 16-85 years, respectively. At the time of writing this paper, there were no recent nationally representative statistics in Australia (ie, statistics published after 2008) regarding the prevalence of *subclinical* social anxiety. Despite changes to SAD diagnostic criteria in the Diagnostic and Statistical Manual of Mental Disorders, fifth edition [1] since the National Comorbidity Survey Replication and NSMHWB, the prevalence of SAD is considered unlikely to have changed [12].

Approximately half of people with mental health problems (eg, SAD) do not receive treatment for 15 years [1]. Early access to empirical information, clinical assessment, and efficacious treatment has the potential to decrease the severity and pervasiveness of social anxiety. Ubiquitous commercial mobile apps may be helpful if used adjunctively with psychoeducational interventions that provide educational materials, screening assessments, feedback, or advice regarding treatment [13]. Commercial mobile apps are developed by commercial, *for-profit* organizations but are not necessarily paid apps as many contain advertising. Psychoeducational apps have the potential to empower people, promote positive behaviors, facilitate personal symptom management, and enhance communication between clients and mental health professionals [14-16].

We identified no published empirical research specifying the reasons why people use or do not use social anxiety apps. However, 2 studies on health apps in the United States provide some insight. A web-based survey of 811 people showed the importance people place in considering content, ease of use, cost, encryption, responsive features, customization, privacy policy, and research evidence [17]. A cross-sectional survey identified that iPhone (iOS operating system) and Samsung (Android operating system) smartphone users stop using apps because they find them to be boring or they do not trust how their personal information will be used or managed. People also stop using health apps because it is burdensome to enter data, they become disengaged, or there are hidden costs [18].

At Stockholm University, academics developed an app called *Challenger* [19], which was available for iPhones only in Sweden’s Apple App Store. The app includes several customizable features to enhance user engagement during internet-based cognitive behavioral therapy (CBT), such as gamification (eg, challenging board games with self-care rewards), personal skills training, goal setting, social interactions between end users, and notifications. Given the strong evidence base for CBT in treating anxiety disorders [20], Challenger has the potential to substantially enhance the ongoing management of social anxiety.

Of 52 anxiety apps, 2 social anxiety apps, including psychological techniques, were identified in a systematic review of the Romanian Apple App Store and Google Play Store (although app and developer names were not published) [21]. The top features of the anxiety apps included text, audio, worksheets, diaries, and animations. The main psychotherapeutic techniques suggested by these apps include progressive muscle relaxation, breathing, and emotional regulation. Two-thirds of the apps were free to download from the app stores; the remainder were between US $0.99 and $8.71 in price.

In another study, 7 psychoeducational and exclusively social anxiety apps in the New Zealand iTunes Store (now Apple App Store), Google Play Store, and Windows Store (now Microsoft Store) were reviewed [22]. Most of these psychoeducational apps were not universally accessible across mobile platforms and devices (ie, Apple [iOS], Google [Android OS], and Microsoft [Windows OS] smartphones and tablets). None contained expert information, had an evaluation of effectiveness published, or were developed by medical or not-for-profit institutions. Although specific psychoeducational apps were not identified in the published article, the names and platforms of all apps reviewed have been provided. Given that most apps are not accessible to people in Australia, it is difficult to substantiate these research findings. Further, the apps in this study were not physically downloaded and reviewed individually, meaning limited conclusions can be drawn regarding the content, purpose, and media within the apps.

The objectives of this study were to (1) review the social anxiety apps in the two most popular Australian mobile app stores (ie, Apple App Store and Google Play Store); (2) report the content, technical properties, and descriptive features of the apps, including their platforms, purpose, and media; (3) evaluate psychoeducational app quality using the Mobile App Rating Scale (MARS) [23]; and (4) recommend the use of any quality, evidence-based apps to others who may benefit in the community.

### What This Paper Adds

This paper makes a significant contribution to the limited academic literature and informs potential end users, mobile app developers, mental health clinicians, and academics about the content and quality of psychoeducational social anxiety apps. To our knowledge, this is the first peer-reviewed study to report the quality evaluation findings of commercial psychoeducational social anxiety apps. We are also the first to publish findings regarding the purposes, mobile platforms, and media of social anxiety apps available for download from Australian app stores. Our study uses an existing methodological framework previously used by New Zealand [22] and Australian [24] researchers to assess the quality of psychoeducational social anxiety apps available in Australia. Our research findings could inform mobile app developers of ways to improve the design, features, and content of social anxiety apps and avoid potential technological problems before development begins. Empirically informed, high-quality apps could then lead to enhanced user engagement, knowledge about social anxiety, increased help-seeking behavior, earlier treatment, and improved mental health.

## Methods

### Overview

The systematic app search and evaluation described in this paper focused on readily available *commercial* psychoeducational *social anxiety* mobile apps in Australia for smartphones and tablet devices. Therefore, apps were excluded if they were only accessible to specific organizations or clinical research participants; for example, an app in the United States, which is the only app we identified to be clinically proven to reduce social anxiety [25], was excluded because it was only accessible to study participants in that country during the randomized controlled trial.

### Design

#### Overview

This systematic stepwise app search and evaluation was informed by the methodology of 2 studies: one investigated commercial social anxiety apps in New Zealand, and the other evaluated the quality of medication adherence apps in Australia [22,24]. The app search and selection strategy in our study was guided by the PRISMA (Preferred Reporting Items for Systematic Reviews and Meta-Analyses) [26].

The key steps in our study involved (1) systematically searching for, identifying, and selecting psychoeducational social anxiety apps in the Australian Apple App Store and Google Play Store; (2) downloading, using, and reviewing relevant apps; (3) reporting technical and descriptive information available in the app stores, an online app warehouse, and individual apps; (4) evaluating app quality using the MARS [23]; and (5) deciding whether to recommend apps to end users. Data were managed using Microsoft Excel spreadsheets (Version 16.27 for Mac).

**Figure 1:**
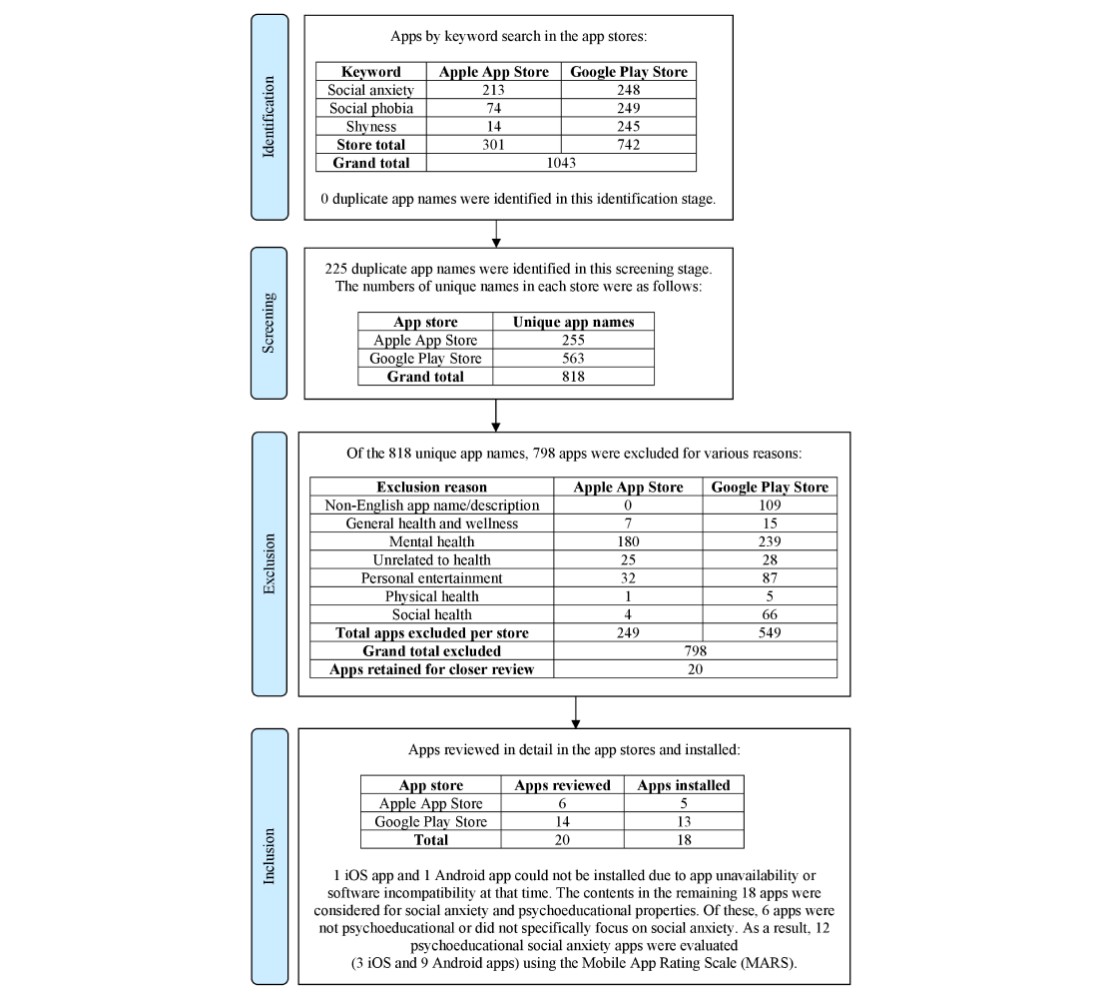
PRISMA (Preferred Reporting Items for Systematic Reviews and Meta-Analyses) flowchart: app search, review, and selection process.

Searches across all categories in the app stores occurred between August 13 and 25, 2019. Depending on the operating system required for app compatibility, apps were downloaded to an Apple iPad (iOS version 12.3.1, sixth Generation MR7F2X/A) or Samsung Galaxy tablet (Android version 4.4.4, SM-T560). At that time, the most recent software packages were installed for these devices before apps were downloaded and installed. The four steps of the app search, review, and selection process, outlined in Figure 1, included app identification, screening, exclusion, and inclusion.

#### Step 1: App Identification

TEH conducted 3 separate keyword searches in 2 app stores for app names and descriptions containing the keywords *social anxiety*, *social phobia,* and *shyness*, which yielded 6 keyword groups. The exact app names and version numbers identified (if available) for each of the 6 search groups are listed in Multimedia Appendix 1. The app operating systems, keywords searched, and dates of data extraction have been provided. Owing to character limits of app names in the Apple App Store, the full names of 34 iOS apps were identified by web search using partial app names and other identifying information provided in the stores. English app names were then sorted alphabetically (numbers and special characters [eg, # and %] excluded), and then app names in all languages were compared and counted for each group.

#### Step 2: App Screening

Data from all groups in Multimedia Appendix 1 were integrated into Multimedia Appendix 2. Columns were added to identify app stores and duplicate app names. English app names were then sorted alphabetically across the 2 app stores. Excel conditional formatting highlighted duplicate app names, which were subsequently reviewed. App names, versions, and developers were considered in determining duplicates. The number of duplicates and unique apps was added up for each app store and overall.

#### Step 3: App Exclusion

Individual app names, descriptions, languages, purposes, intended audiences, screenshots, and relevant videos in the app stores were reviewed. Exclusion categories were developed based on the World Health Organization’s definition of health [27] and the American Psychiatric Association’s key SAD diagnostic criteria [1], outlined earlier in this paper. According to the World Health Organization, “health is a state of complete physical, mental and social well-being and not merely the absence of disease or infirmity” [27]. Therefore, apps were excluded if they did not have an English name or description; were completely unrelated to health; did not have a specific focus on social anxiety but focused more generally on health and wellness, mental health, physical health, or social health; or if they were intended for personal entertainment. The number of apps excluded for these reasons was counted in each app store and added up overall. The reasons for exclusion for the apps reviewed during step 3 are provided in Multimedia Appendix 2.

#### Step 4: App Inclusion

The remaining apps were reviewed by viewing app store websites and an app clearing warehouse website, specifically, app images, detailed descriptions, and related commentaries. Psychoeducational apps and apps targeting social anxiety are identified in Multimedia Appendix 2. The apps evaluated using the MARS [23] are listed in Multimedia Appendix 3, including their names, version numbers (if available), platforms, and associated websites.

### Collection of App Technical and Descriptive Information

The App Classification section of the MARS [23] was completed for each mobile app evaluated. Although most information was obtained from the app stores and individual apps, some information was collected by searching various app stores and an app warehouse website. Additional information was collected for each app and is available in Multimedia Appendix 2, including the purpose (eg, multipurpose, self-assessment, supportive resources, or therapeutic treatment) and media type (eg, text and audio, text and visual, or text only).

### Evaluating Apps Using the MARS

The MARS [23] consists of an *app quality rating* scale (sections A-D), an *app subjective quality* scale (section E), and an *app-specific* scale (section F). The app quality rating scale assesses various dimensions of app quality, including engagement (section A), functionality (section B), esthetics (section C), and information (section D). The 19 items of the scale were rated on a 5-point scale from inadequate to excellent. Sections A to D have an internal consistency of α=.90 and an interrater reliability intraclass correlation coefficient of 0.79. The app subjective quality scale has 4 items with different rating scales that assess whether one would recommend apps to others, how many times apps may be used for a 12-month period, whether one would pay for apps, and overall star ratings. The app-specific scale has 6 items that assess the perceived impact of apps on the user’s awareness, knowledge, attitudes, intention to change, help-seeking behaviors, and actual behavior change.

However, given that the interrater reliability has not been determined for the app subjective quality scale and the app-specific scale, the initial quality assessment of apps in our study was predominantly based on the app quality rating scale. Apps were also assessed using the app subjective quality scale to supplement the findings and provide app developers with additional useful information in developing innovative social anxiety apps. The app-specific scale was not used in our study because the items were out of scope; it would be better suited to assess the perceptions of people with social anxiety (ie, how social anxiety apps could impact their knowledge, attitudes, and behaviors).

Two raters (TEH and SP) with knowledge regarding mobile apps, backgrounds in mental health, and training in using the MARS independently evaluated 12 psychoeducational social anxiety apps. This involved using each app for at least 15 minutes, reviewing information about the apps in the app stores, and then completing both the app quality rating scale and the app subjective quality scale. After evaluating all apps, TEH and SP discussed their individual ratings for the individual apps, particularly ratings that were substantially different (ie, 2 or more points different on the rating scales). This consultation stage ensured that key aspects of the apps and different opinions were considered before finalizing app ratings. Individual app scores of TEH and SP for the app quality rating scale and the app subjective quality scale were manually entered into separate Excel spreadsheets. Scores were averaged overall for each dimension of the app quality rating scale and items of the app subjective quality scale.

## Results

### App Technical and Descriptive Information

A total of 1043 apps were identified in the mobile app stores, of which 12 (1.15%) psychoeducational social anxiety apps were included for evaluation (Apple App Store=301 apps, 3 included for evaluation; Google Play Store=742 apps, 9 included for evaluation). Figure 1 presents the PRISMA flowchart for progress through the selection process and demonstrates why only 12 apps were downloaded and evaluated using the MARS.

The updated version numbers and exact production dates were available for 11 apps. Most were updated between 2016 and 2018. The earliest app was first released in 2011. The most recently updated apps in 2018 and 2019 were downloaded from the Google Play Store. The apps in the Apple App Store were updated during 2016 and 2017.

At the time of download, all apps were affiliated with commercial developers, as opposed to not-for-profit organizations. Only 5 were rated by app users (1 was rated by an Apple app user; 4 were rated by Android users), which had an overall mean rating of 4.2 out of 5 stars (SD 0.7). Most apps (n=9) targeted all age groups; however, 3 targeted adolescents and adults. In total, 9 apps were free to download. An app in the Apple App Store was priced at US $3.32 (Aus $4.49). The prices of the 2 other apps in the Google Play Store were US $2.95 (Aus $3.99) and US $3.69 (Aus $5.00).

All 12 apps were psychoeducational; however, their intended purposes differed. Five apps were for multiple purposes, 1 was for self-assessment, 3 contained supportive resources, and 3 had a therapeutic aim.

Similarly, the media of each app differed markedly. Of the 12 apps evaluated, 25% (3) were text and audio, 42% (5) were text and visual, and 33% (4) were text only. All apps contained advertisements for various products and services. App technical and descriptive information, including content focus, theoretical background, and therapeutic strategies, are listed in Multimedia Appendix 4.

### App Quality Evaluation

#### MARS App Quality Rating Subscale (Sections A-D)

The top 5 ranked apps using the MARS included 3 Apple apps and 2 Android apps. From highest to lowest quality, they included *Beat Social Phobia with Andrew Johnson*, *Social Anxiety Test* (Mood Tools), *Social Anxiety Test* (Eddie Liu), *Social Anxiety Test-Psychological Test* and *How to Overcome Shyness* (Iaks Solutions). Individual and mean app quality ratings for each item in sections A to D are provided in Multimedia Appendix 5. The mean quality ratings of the apps are presented in Table 1.

The measures of central tendency (mean, median, and mode) and dispersion (SD and range) of overall app quality and the four app dimensions are presented in Table 2.

**Table 1:** Mean app quality ratings (including the 4 dimensions of engagement, functionality, esthetics, and information)^a^.

App name	Operating system	Quality	Engagement	Functionality	Esthetics	Information
Beat Social Phobia with Andrew Johnson	iOS	4.10	4.40	4.50	3.67	3.83
Social Anxiety Test (Mood Tools)	Android	4.07	3.20	4.75	4.33	4.00
Social Anxiety Test (Eddie Liu)	iOS	3.94	3.00	4.50	4.00	4.25
Social Anxiety Test - Psychological Test	iOS	3.56	2.70	4.00	4.17	3.38
How To Overcome Shyness (Iaks Solutions)	Android	3.54	3.10	4.38	3.33	3.33
Beat Social Phobia	Android	3.33	3.60	3.88	2.83	3.00
Social Anxiety Disorder (Afradad Media)	Android	3.25	2.00	3.88	3.83	3.30
Social Anxiety Hypnosis	Android	3.10	2.20	4.13	2.33	3.75
How To Overcome Shyness (The Almighty Dollar)	Android	3.09	2.40	3.63	3.33	3.00
How To Overcome Shyness (Dierre09)	Android	2.75	1.90	4.00	2.50	2.60
Social Anxiety Disorder (Bedieman)	Android	2.69	1.70	3.63	2.17	3.25
Recognize Social Anxiety Disorder	Android	2.49	1.60	3.63	2.50	2.25

^a^Developer names are in parentheses for apps that share the same name.

**Table 2:** Measures of central tendency and dispersion of aggregate app quality ratings (including the four dimensions of engagement, functionality, esthetics, and information).

Variable	Quality	Engagement	Functionality	Esthetics	Information
Value, mean (SD)	3.33 (0.54)	2.65 (0.84)	4.07 (0.38)	3.25 (0.76)	3.33 (0.58)
Value, median (range)	3.29 (4.10-2.49)	2.55 (4.40-1.60)	4.00 (4.75-3.63)	3.33 (4.33-2.17)	3.32 (4.25-2.25)
Value, mode	N/A^a^	N/A	3.63	2.50 and 3.33^b^	3.00

^a^N/A: not applicable.

^b^Bimodal.

Regarding the central tendency measures for the apps, the quality ratings were mean 3.33 (SD 0.54) and median 3.29; there was no mode. These ratings showed that the quality of the apps according to the MARS was acceptable. The measures of dispersion demonstrated the similarity between app quality in Australian app stores. The highest-ranked app had a quality score of 4.10 and the lowest-ranked app had a score of 2.49 (range 1.61).

These results indicated that the strongest determinant of higher quality social anxiety apps was their functionality, as evidenced by a mean rating of 4.07 (SD 0.38). Specifically, most apps scored positively for performance, ease of use, and responsive taps, swipes, and scrolls (gestural design). Most components and functions of the top 5 social anxiety apps functioned correctly. These apps were relatively easy to use because of their clear labels, icons, and instructions.

Poorer app quality ratings were recorded for end user engagement, specifically for entertainment, customization, and interactivity. These results suggest that commercial psychoeducational social anxiety apps are not particularly fun to use. None of the apps evaluated in our review used gamification to entertain end users. Furthermore, most of these apps do not allow users to customize settings and preferences for app features, such as sound, content, and notifications.

#### MARS App Subjective Quality Subscale (Section E)

The mean subjective quality app ratings in Tables 3 and 4 are similar to the mean app quality ratings in Tables 1 and 2.

**Table 3:** Mean subjective quality ratings of the apps^a^.

App name	Operating system	Subjective quality	Recommendation	12-month usage	Payment	Star rating
Beat Social Phobia with Andrew Johnson	iOS	3.88	4.00	3.50	4.00	4.00
Social Anxiety Test (Eddie Liu)	iOS	3.50	4.00	3.50	3.00	3.50
Social Anxiety Test (Mood Tools)	Android	3.50	4.00	3.50	3.00	3.50
Social Anxiety Hypnosis	Android	3.38	3.50	3.50	3.00	3.50
How To Overcome Shyness (Iaks Solutions)	Android	2.75	3.00	3.00	2.00	3.00
Beat Social Phobia	Android	2.50	3.00	3.00	1.00	3.00
Social Anxiety Disorder (Afradad Media)	Android	2.25	2.50	2.50	1.00	3.00
How To Overcome Shyness (The Almighty Dollar)	Android	2.13	2.50	2.50	1.00	2.50
Social Anxiety Disorder (Bedieman)	Android	2.00	2.50	2.00	1.00	2.50
Recognize Social Anxiety Disorder	Android	1.50	1.50	1.50	1.00	2.00
Social Anxiety Test - Psychological Test	iOS	1.50	2.00	1.50	1.00	1.50
How To Overcome Shyness (Dierre09)	Android	1.38	1.50	1.50	1.00	1.50

^a^Developer names are in parentheses for apps that share the same name.

**Table 4:** Measures of central tendency and dispersion for app aggregate subjective quality ratings.

Variable	Subjective quality	Recommendation	12-month usage	Payment	Star rating
Value, mean (SD)	2.52 (0.88)	2.83 (0.91)	2.63 (0.83)	1.83 (1.11)	2.79 (0.81)
Value, median (range)	2.38 (3.88-1.38)	2.75 (4.00-1.50)	2.75 (3.50-1.50)	1.00 (4.00-1.00)	3.00 (4.00-1.50)
Value, mode	1.50 and 3.50^a^	2.50 and 4.00^a^	3.50	1.00	3.00 and 3.50^a^

^a^Bimodal.

The app subjective quality ratings for section E of the MARS are available in Multimedia Appendix 6.

Of the top 5 apps, 4 (identified in Table 1) were rated moderately in terms of app subjective quality. From the highest- to the lowest-ranked app, they included *Beat Social Phobia with Andrew Johnson*, *Social Anxiety Test* (Eddie Liu), *Social Anxiety Test* (Mood Tools), *Social Anxiety Hypnosis*, and *How to Overcome Shyness* (Iaks Solutions). The mean app subjective quality of the 12 apps was 2.52 (SD 0.88), the median was 2.38, and there were 2 modes (1.50 and 3.50). These measures of central tendency showed that most of the apps’ subjective quality ratings were similar and centered around the mean. The measures of dispersion, including the range of 2.50 (3.88-1.38), showed little variability between the subjective quality scores.

The app subjective quality ratings showed that independent raters (TEH and SP) would consider recommending 50% (6/12) of the psychoeducational apps to others, based on their personal experiences (mean 2.83, SD 0.91). However, none of the apps would definitely be recommended, and given that this measure has not been tested for validity and consistency, we cannot professionally recommend that others use the apps. Raters (TEH and SP) would consider using 50% (6/12) of these apps once in a 12-month period if they were relevant to their needs and wants (mean 2.63, SD 0.83). However, raters typically did not want to pay for the apps (mean 1.83, SD 1.11). Most apps received a star rating of at least 3 out of 5 (mean 2.79, SD 0.81).

## Discussion

### Overview

In our discussion, we outline the principal research results, considering our research objectives, which were to review the psychoeducational social anxiety mobile apps in the Australian Apple App Store and Google Play Store; describe the apps, their platforms, purpose, and media; evaluate the apps using the MARS [23]; and recommend the use of any quality evidence-based apps to others. We describe the problems encountered when researching psychoeducational social anxiety apps and challenge commercial app developers to enhance existing apps and design new apps that meet users’ needs and wants. App development opportunities include enhancing the efficiency of locating social anxiety apps in app stores, improving international access to apps, and decreasing app-device incompatibilities. Further, we discuss descriptive and technical considerations for psychoeducational social anxiety apps, the lack of empirical evidence, and some potential limitations of mobile app reviews.

### Principal Findings

There was a large number of apps available in the app stores, and they varied in quality. It is of interest that on the MARS [23], the range of scores between the top 5 and lowest quality apps was greater for the app subjective quality scale (section E) than the app quality rating scale (sections A-D). Considering all MARS ratings in our study, the 3 highest quality apps were *Beat Social Phobia with Andrew Johnson*, *Social Anxiety Test* (Mood Tools), and *Social Anxiety Test* (Eddie Liu).

The difference in app ratings between the 2 subscales of the MARS could be attributable to scale design and the type and number of questions in each subscale. For example, the app subjective quality scale consists of 4 items, each with different rating scales. Averaging scores across these 4 items makes it challenging to interpret the results. The app quality rating scale has a higher level of face validity because it taps into a wider range of app dimensions, as opposed to only user preferences and potential future actions.

However, there is more to consider when evaluating apps than features and content. For instance, none of the social anxiety apps evaluated in this study appear to have been designed based on empirical evidence, and none have been evaluated to determine clinical effectiveness. App end users should provide constructive feedback to developers by contacting them directly (eg, by locating their contact details in the app stores), providing star ratings, and writing informative and honest reviews in the app stores. Similarly, app developers should work collaboratively with potential end users and clinical populations to identify and best meet their needs and desires.

### Challenges for Commercial Social Anxiety App Developers

Our results highlighted the challenges in locating good-quality psychoeducational apps focused specifically on social anxiety in Australian mobile app stores. The initial search of the Google Play Store and Apple App Store revealed 1043 app names, based on keywords in app names and descriptions selected for their apparent relevance to social anxiety, thus representing the type of keywords consumers might use. However, the hit rate (*true positives*) for psychoeducational social anxiety apps was only 1.15% (12/1043). This poor hit rate is consistent with a review of 1154 apps in the New Zealand mobile app stores, which identified 13 psychoeducational social anxiety apps [22] with a hit rate of 1.13%. As the proportion of misses (*false positives*) exceeds 98% in both studies, the keywords in social anxiety app names and descriptions need to be improved. Unlike researchers and app developers, other app users do not typically have the time and patience to run systematic searches for relevant apps and often do not have knowledge regarding how to identify all relevant apps in the app stores.

Access to commercial psychoeducational social anxiety apps is limited because of end users’ geographic locations. On the basis of the findings of the New Zealand review [22], only 2 of the 12 apps in our research were available in June 2016 in the New Zealand Google Play Store and iTunes Store (potentially different versions). It is noteworthy that in Australia, we were unable to download apps from the New Zealand Google Play Store and Apple App Store. The social anxiety apps identified by our New Zealand colleagues include *Social Anxiety Hypnosis* and *Beat Social Phobia with Andrew Johnson*. Developers, legislators, and intellectual property regulators should consider opening up web-based markets to increase access to apps. This is particularly important for well-conceptualized psychoeducational social anxiety apps. For example, *Challenger*, which was developed at Stockholm University, is only available in Sweden’s Apple App Store [19].

Psychoeducational social anxiety apps are less common for Apple iPhones and iPads than for Android smartphones and tablets. In our study, only 25% (3/12) of the apps could be used on iOS devices, compared with 75% (9/12) of the apps for Android devices. Only 2 apps were available for both Apple and Android devices, namely, *Beat Social Phobia* and *Social Anxiety Test*. This has created an additional barrier for people with Apple devices to access psychoeducational social anxiety apps. Although the Android software for apps was relatively current at the time of review, iOS software for the Apple apps was updated 3 years before the review. Considering potential app-device incompatibility problems, people may have difficulty in downloading and accessing apps on newer, recently updated iPhones and iPads. However, given that 9 of the apps were free and the other 3 were reasonably priced, according to our standards, it is unlikely that there are financial barriers to access the apps.

### Descriptive and Technical Considerations

The technical properties and descriptive content of the psychoeducational social anxiety apps, including their platforms, purposes, and media, have been described in the *Results* section of this paper. However, we believe it is important to highlight the need for empirically informed apps and reliable app quality assessment tools.

The apps reviewed in our study were intended to be psychoeducational; however, most contained outdated information about social anxiety and did not appear to be informed by empirical research evidence. Similar to earlier findings [22], there is no evidence that social anxiety apps in Australian app stores were developed by reputable not-for-profit institutions. All apps evaluated in our research were developed by commercial, *for-profit* organizations and contained advertising to promote often unrelated products and services. Although advertising may be included in mobile apps for financial gain, it may also be necessary to cover the costs of developing and maintaining apps, as app development can be expensive, and funding is limited. Further, the social anxiety apps contained insufficient information to confirm whether the content was based on expert knowledge and experience.

*Challenger* is the only publicly available, empirically-based psychoeducational social anxiety app we identified in our literature review [19]. On the basis of the authors’ description of Challenger’s features and technical specifications, we could not identify any social anxiety apps in Australian app stores of comparable quality. Raters TEH and SP were unable to evaluate the quality of Challenger using the MARS because the app was not available for download from the Australian app stores. Although the clinical effectiveness of Challenger in managing social anxiety has not been evaluated, it is the first commercial social anxiety app that uses gamification, goal setting, and CBT to engage end users. App developers should consider Challenger’s technical and descriptive features, particularly when conceptualizing new evidence-based and empirically evaluated apps.

The completion rate, validity, and reliability of app store reviews in terms of determining app quality are questionable. Only 41.7% (5/12) of the social anxiety apps evaluated in our research were reviewed and rated by Apple App or Google Play Store app users. The moderated reviews in the app stores allow users to provide star ratings and post comments about the apps. However, only 2 of the 5 apps rated to be the highest in quality in our study (out of the 12 apps) received high-quality app store ratings in the app stores. For example, the average MARS app *quality* ratings in our study for *Beat Social Phobia with Andrew Johnson* (mean 4.10) and *Social Anxiety Test* (Mood Tools) (mean 4.07) were similar to the Apple App Store and Google Play Store, with ratings of 5 and 4.5 stars, respectively. As these apps have been available in the Australian app stores for several years and have been downloaded more than 10,000 times, we expected more than a few dozen reviews. Further, most app store reviews consisted only of star ratings rather than ratings and comments; therefore, it was not possible to verify quantitative ratings with qualitative data.

One potential explanation for these varied findings could be the different backgrounds (socioeconomic, cultural, and linguistic), perceptions, and expectations of Australian app users. Although the app raters TEH and SP are highly experienced in using mobile health apps and work in mental health settings, others in the Australian community may not be as objective and could find it difficult to provide detailed feedback when reviewing mobile apps. Furthermore, unlike the mobile app stores which allow app users to provide general written feedback, the items in the MARS allow raters to focus on specific aspects of apps, thereby allowing for more rigorous evaluation. Another potential reason for the varied quality ratings between the app stores and the MARS could be that raters (TEH and SP) reviewed all psychoeducational social anxiety apps in the two most popular Australian app stores. People in the community may be more selective in providing positive feedback for apps if they are more engaged app users (people who are disengaged may simply delete the app). It is also possible that the small number of web-based app reviews could be related to commercial developers wanting to positively market their apps to increase downloads and, therefore, advertising revenue. However, it is important to note that this comment is based on anecdotal feedback from app users and the professional experiences of the app raters (TEH and SP).

### Strengths and Limitations

This is the first peer-reviewed systematic app review in which a published search strategy [26] was used to identify commercial social anxiety mobile apps in Australia. To the best of our knowledge, no other study has evaluated the quality of commercial social anxiety apps using a validated assessment inventory. The MARS is a suitable and useful tool for the purpose of quality assessment of social anxiety apps. However, mobile app raters need to have a good understanding of the terminology of the MARS items and substantial experience in using apps. The findings in our study would be very useful for the public in identifying suitable social anxiety apps and for developers in designing high-quality apps that are engaging, functional, esthetically pleasing, and contain appropriate information.

As our study involved reviewing the two most popular app stores in Australia, we believe that most of the psychoeducational social anxiety apps in Australia have been reviewed. However, the Apple App Store and Google Play Store have limited search parameters, making it challenging to identify relevant apps using keywords alone. Although our research findings are relevant to international app developers, they may only be directly transferrable to smartphone and tablet apps available in those app stores. We also acknowledge that other psychoeducational social anxiety apps are available, such as Microsoft, Amazon, Aptoide, F-Droid, and AppBrain apps.

### Conclusions and Future Recommendations

The psychoeducational social anxiety apps available for download from the Australian Apple App Store and Google Play Store are of acceptable quality, either free or inexpensive to access, and contain some useful features and content that may assist people experiencing social anxiety or those who know someone with social anxiety. However, these apps appear to contain substantial amounts of outdated and nonempirical information, which is concerning given there is no evidence to suggest that they are practically useful in managing social anxiety. These apps could potentially cause harm to end users by indirectly or directly encouraging people to self-diagnose psychiatric disorders using web-based inventories (which were developed more than 20 years ago) and make personal decisions based on anecdotal evidence and untested treatment options.

Further work should focus on the development of tailored apps to meet the needs and desires of end users through researchers and app developers working collaboratively with mental health professionals, people with social anxiety, and people who know others with social anxiety. Information gleaned from co-design workshops, interviews, focus groups, web-based panels, and evidence-based information from peer-reviewed academic research could lead to the development of app prototypes that are high in quality; contain best practice strategies to manage social anxiety; and designed with suitable data collection, security, and sharing capabilities. Data collection options to better understand social anxiety could include (1) optimizing validated screening tools (eg, questionnaires and inventories) by considering internet and social media use, (2) mental health tracking systems to allow for efficacy testing, and (3) qualitative data collection tools to understand end users’ experiences, for example, personal diaries and open-ended questionnaires.

Furthermore, it is time consuming to locate all the potential psychoeducational social anxiety apps in popular app stores, which could potentially increase stress and anxiety for those who are trying to find the right app for their circumstances. App developers could enhance the ease of locating apps by incorporating keywords more specific to social anxiety in app descriptions and names and avoiding broad keywords that encompass mental health problems more generally.

Finally, app compatibility can be enhanced across several mobile app platforms (not just iOS and Android devices). App developers could consider designing hybrid apps to be used across different devices and operating systems or separate versions of the same native app to be downloaded to devices with specific operating systems.

## Appendix

Multimedia Appendix 1 Apps searched by keyword, device, and app store.

Multimedia Appendix 2 App list per app store and exclusion reasons.

Multimedia Appendix 3 Psychoeducational social anxiety apps evaluated.

Multimedia Appendix 4 App technical and descriptive information.

Multimedia Appendix 5 Quality ratings (sections A-D) aggregate and individual raters.

Multimedia Appendix 6 Subjective ratings (section E): aggregate and individual raters.

## Abbreviations

CBT: cognitive behavioral therapy
MARS: Mobile App Rating Scale
NSMHWB: National Survey of Mental Health and Wellbeing
PRISMA: Preferred Reporting Items for Systematic Reviews and Meta-Analyses
SAD: social anxiety disorder

## References

[1] American Psychiatric Association (2013). Diagnostic and Statistical Manual of Mental Disorders, Fifth Edition.

[2] United States National Institute of Mental Health (2016). Social Anxiety Disorder: More Than Just Shyness.

[3] Buist-Bouwman MA, De Graaf R, Vollebergh WAM, Alonso J, Bruffaerts R, Ormel J (2006). Functional disability of mental disorders and comparison with physical disorders: a study among the general population of six European countries. Acta Psychiatr Scand.

[4] Van Ameringen M, Mancini C, Farvolden P (2003). The impact of anxiety disorders on educational achievement. J Anxiety Disord.

[5] Russell G, Topham P (2012). The impact of social anxiety on student learning and well-being in higher education. J Ment Health.

[6] Grant BF, Hasin DS, Blanco C, Stinson FS, Chou SP, Goldstein RB, Dawson DA, Smith S, Saha TD, Huang B (2005). The epidemiology of social anxiety disorder in the United States: results from the National Epidemiologic Survey on Alcohol and Related Conditions. J Clin Psychiatry.

[7] Fehm L, Beesdo K, Jacobi F, Fiedler A (2008). Social anxiety disorder above and below the diagnostic threshold: prevalence, comorbidity and impairment in the general population. Soc Psychiatry Psychiatr Epidemiol.

[8] United Nations Department of Economic and Social Affairs (2019). World Population Prospects 2019.

[9] American Psychiatric Association (2000). Diagnostic and Statistical Manual of Mental Disorders (DSM-IV-TR).

[10] Kessler RC, Chiu WT, Demler O, Merikangas KR, Walters EE (2005). Prevalence, severity, and comorbidity of 12-month DSM-IV disorders in the National Comorbidity Survey Replication. Arch Gen Psychiatry.

[11] Australian Bureau of Statistics (2008). National Survey of Mental Health and Wellbeing 2007: Summary of Results.

[12] Crome E, Grove R, Baillie AJ, Sunderland M, Teesson M, Slade T (2015). DSM-IV and DSM-5 social anxiety disorder in the Australian community. Aust N Z J Psychiatry.

[13] Donker T, Griffiths KM, Cuijpers P, Christensen H (2009). Psychoeducation for depression, anxiety and psychological distress: a meta-analysis. BMC Med.

[14] Coles ME, Coleman SL (2010). Barriers to treatment seeking for anxiety disorders: initial data on the role of mental health literacy. Depress Anxiety.

[15] Olfson M, Guardino M, Struening E, Schneier FR, Hellman F, Klein DF (2000). Barriers to the treatment of social anxiety. Am J Psychiatry.

[16] Chartier-Otis M, Perreault M, Bélanger C (2010). Determinants of barriers to treatment for anxiety disorders. Psychiatr Q.

[17] Schueller SM, Neary M, O'Loughlin K, Adkins EC (2018). Discovery of and interest in health apps among those with mental health needs: Survey and focus group study. J Med Internet Res.

[18] Krebs P, Duncan DT (2015). Health app use among US mobile phone owners: A national survey. JMIR Mhealth Uhealth.

[19] Miloff A, Marklund A, Carlbring P (2015). The challenger app for social anxiety disorder: New advances in mobile psychological treatment. Internet Interventions.

[20] Hofmann SG, Asnaani A, Vonk IJJ, Sawyer AT, Fang A (2012). The efficacy of cognitive behavioral therapy: A review of meta-analyses. Cognit Ther Res.

[21] Sucala M, Cuijpers P, Muench F, Cardoş R, Soflau R, Dobrean A, Achimas-Cadariu P, David D (2017). Anxiety: There is an app for that. A systematic review of anxiety apps. Depress Anxiety.

[22] Alyami M, Giri B, Alyami H, Sundram F (2017). Social anxiety apps: A systematic review and assessment of app descriptors across mobile store platforms. Evid Based Ment Health.

[23] Stoyanov SR, Hides L, Kavanagh DJ, Zelenko O, Tjondronegoro D, Mani M (2015). Mobile app rating scale: A new tool for assessing the quality of health mobile apps. JMIR Mhealth Uhealth.

[24] Santo K, Richtering SS, Chalmers J, Thiagalingam A, Chow CK, Redfern J (2016). Mobile phone apps to improve medication adherence: A systematic stepwise process to identify high-quality apps. JMIR Mhealth Uhealth.

[25] Enock PM, Hofmann SG, McNally RJ (2014). Attention bias modification training via smartphone to reduce social anxiety: A randomized, controlled multi-session experiment. Cogn Ther Res.

[26] Moher D, Liberati A, Tetzlaff J, Altman DG (2009). Preferred reporting items for systematic reviews and meta-analyses: The PRISMA statement. PLoS Med.

[27] World Health Organization (1946). Preamble to the constitution of WHO. World Health Organization.

