# Supplementary material for: Psychoeducational Social Anxiety Mobile Apps: Systematic Search in App Stores, Content Analysis, and Evaluation
Source: JMIR Mhealth Uhealth. 2021 Sep 21;9(9):e26603. doi: 10.2196/26603 (PMC8493451; doi:10.2196/26603)
Supplement: Multimedia Appendix 3 [file mhealth_v9i9e26603_app3.docx]

#### Multimedia Appendix 3

#### Psychoeducational social anxiety apps evaluated

| **App name** | **Version** | **Platform** | **Website** |
| --- | --- | --- | --- |
| Beat Social Phobia with Andrew Johnson^^^ | 8.38 | iPad | <https://apps.apple.com/au/app/beat-social-phobia-with-andrew-johnson/id321996942> |
| Social Anxiety Test (Eddie Liu)^*^ | NA | iPad | <https://apps.apple.com/au/app/social-anxiety-test/id1244492208> |
| Social Anxiety Test - Psychological Test | 1.1 | iPad | <https://apps.apple.com/jo/app/social-anxiety-test-psychological-tests/id1128751446> |
| Beat Social Phobia^^^ | 5.0.1 | Android Tablet | <https://play.google.com/store/apps/details?id=com.hivebrain.andrewjohnson.phobia2> |
| How To Overcome Shyness (Iaks Solutions) | 1.0 | Android Tablet | <https://play.google.com/store/apps/details?id=how.to.overcome.shyness> |
| How To Overcome Shyness (dierre09) | 2.0 | Android Tablet | <https://m.apkpure.com/how-to-overcome-shyness/com.chj1howtoovercomeshyness> |
| How To Overcome Shyness (The Almighty $) | 1.0 | Android Tablet | <https://play.google.com/store/apps/details?id=com.tadapps.how.to.overcome.shyness> |
| Recognize Social Anxiety Disorder | 3 | Android Tablet | <https://play.google.com/store/apps/details?id=mediaclinic.recognizesocialanxietydisorder> |
| Social Anxiety Disorder (Bedieman) | 1.0.0 | Android Tablet | <https://play.google.com/store/apps/details?id=com.bedieman.socialAnxietydisorder> |
| Social Anxiety Disorder (Afradad Media) | 1.3.0 | Android Tablet | <https://play.google.com/store/apps/details?id=com.afradadmedia.socialanxiety> |
| Social Anxiety Hypnosis | 1.0 | Android Tablet | <https://play.google.com/store/apps/details?id=com.hypnotransformations.socialanxietycure> |
| Social Anxiety Test^*^ | 1.1 | Android Tablet | <https://play.google.com/store/apps/details?id=com.socialanxiety.test> |

The four app names highlighted above are essentially two apps (as indicated by the same colour – green^^^/yellow^*^), available for both iOS and Android operating systems. Beat Social Phobia^^^ and Social Anxiety Test^*^ were separately evaluated by our independent raters (TEH and SP).

Where the app version number is unavailable, or when apps share the same name, developer names are included in parentheses.
