# Supplementary material for: Psychoeducational Social Anxiety Mobile Apps: Systematic Search in App Stores, Content Analysis, and Evaluation
Source: JMIR Mhealth Uhealth. 2021 Sep 21;9(9):e26603. doi: 10.2196/26603 (PMC8493451; doi:10.2196/26603)
Supplement: Multimedia Appendix 4 [file mhealth_v9i9e26603_app4.docx]

**Multimedia Appendix 4**

App technical and descriptive information

| **App name** | **Platform** | **Version**  **number** | **Developer name** | **Date updated (DD/MM/YYYY)** | **App cost**  **($AUD)** | **Age group** | **App rating**  **Stars (N=)** | **App description** | **Technical aspects** | **App content focus** | **Theoretical background/ strategies** |
| --- | --- | --- | --- | --- | --- | --- | --- | --- | --- | --- | --- |
| Beat Social Phobia | Android Tablet | 5.0.1 | Universal Relaxation | 25/08/2015 | 3.99 | General | 4.5(97) | Strategies for relaxation and for becoming more comfortable in social situations. | Allows sharing (Facebook, Twitter, etc.); has an app community; needs web access to function. | Increase happiness/ wellbeing; mindfulness/ meditation/ relaxation; reduce negative emotions; anxiety/ stress; physical health. | Information/education; advice/tips/strategies/skills training; mindfulness/meditation; relaxation. |
| Beat Social Phobia with Andrew Johnson | iPad | 8.38 | Universal Relaxation | 19/09/2016 | 4.49 | General | 5(1) | Strategies for relaxation and for becoming more comfortable in social situations. | Allows sharing (Facebook, Twitter, etc.); has an app community; send reminders; needs web access to function. | Increase happiness/ wellbeing; mindfulness/ meditation/ relaxation; reduce negative emotions; anxiety/ stress; physical health. | Information/education; advice/tips/strategies/skills training; mindfulness/meditation; relaxation. |
| How To Overcome Shyness | Android Tablet | 2.0 | Dierre09 | 08/04/2016 | Free | General | 3.5(6) | Contains an introduction, which contains some general information and statistics about social anxiety. The app is compartmentalized into three different chapters, which contain tips for overcoming social anxiety. | - | Anxiety/stress. | Information/education; advice/tips/strategies/skills training. |
| How To Overcome Shyness | Android Tablet | 1.0 | Iaks Solutions | 19/12/2018 | Free | General | - | Combines information for shyness with social anxiety; provides tips for dealing with social anxiety, and other informative information in the sections “How to Overcome Shyness and Social Anxiety” and “Best ways to Overcome Social Anxiety”. | Allows sharing (Facebook, Twitter, etc.). | Anxiety/stress. | Information/education; advice/tips/strategies/skills training/CBT – cognitive (thought challenging); ACT; strengths based. |
| How To Overcome Shyness | Android Tablet | 1.0 | The Almighty Dollar | 24/07/2018 | Free | General | - | Integrates information regarding social anxiety and shyness, provides resources to other e-books, and is organized into 12 descriptive sections.  The sections specific to social anxiety are “How to Overcome Shyness and Social Anxiety” and “Best ways to Overcome Social Anxiety”. | Allows sharing (Facebook, Twitter, etc.); needs web access to function. | Anxiety/stress. | Information/education; advice/tips/strategies/skills training. |
| Recognize Social Anxiety Disorder | Android Tablet | 3 | Media Clinic | 20/08/2019 | Free | General | - | Information regarding social anxiety disorder, signs and symptoms (cognitive, behavioral, physiological, comorbidity), causes (genetics, social experiences, cultural influences, psychological factors), treatment (psychotherapies, medications), and history. | - | Anxiety/stress. | Information/education. |
| Social Anxiety Disorder | Android Tablet | 1.3.0 | Afradad Media | 28/02/2015 | Free | General | 3.5(33) | Presents information regarding social anxiety in an e-book format.  The e-book has an introduction, conclusion, and is organized into nine other sections informing users regarding the history, etiology, symptoms, and treatment options for social anxiety. | - | Reduce negative emotions; anxiety/stress; behavior change. | Information/education; Advice/tips/strategies/skills training/CBT – cognitive (thought challenging); other (exposure therapy and natural therapies). |
| Social Anxiety Disorder | Android Tablet | 1.0.0 | Bedieman | 03/12/2017 | Free | General | - | Almost identical to the information in and the layout of Media clinic’s Recognize Social Anxiety Disorder app, apart from color and a few images. | - | Anxiety/stress. | Information/education. |
| Social Anxiety Hypnosis | Android Tablet | 1.0 | Kym Tolson and Hani Al-Qasem | 11/10/2011 | 5.00 | General | - | Brief information about social anxiety plus an audio-recording for hypnosis. | - | Increase happiness/ wellbeing; mindfulness/ meditation/ relaxation; anxiety/stress; other (hypnosis). | Information/education; mindfulness/meditation; relaxation. |
| Social Anxiety Test | Android Tablet | 1.1 | Mood Tools | 03/04/2018 | Free | Adolescents; young adults; adults | 4.5(31) | 17-question Social Phobia Inventory [1]; resources regarding social anxiety; online help; access to counselling. | Needs web access to function. | Anxiety/stress; behavior change. | Assessment; feedback; information/education; monitoring/tracking; advice/ tips/ strategies/ skills training; CBT – cognitive (thought challenging). |
| Social Anxiety Test | iPad | NA | Eddie Liu | 2017 (exact date unavailable) | Free | Adolescents; young adults; adults | - | Almost identical to Mood Tool’s Social Anxiety Test app, except for minor color variations, some differences in icon sizes, and a higher image resolution. | Needs web access to function. | Anxiety/stress; behavior change. | Assessment; feedback; information/education; monitoring/tracking; advice/tips/strategies/skills training; CBT – cognitive (thought challenging). |
| Social Anxiety Test - Psychological Test | iPad | 1.1 | Balkanboy Media | 14/04/2017 | Free | Adolescents; young adults; adults | - | 40-question test for social anxiety, score, and description. There is no evidence to suggest that the social anxiety test has been validated. | Needs web access to function. | Anxiety/stress. | Assessment; feedback; information/education. |

**References:**

1. Connor KM, Davidson JR, Churchill LE, Sherwood A, Foa E, Weisler RH. Psychometric properties of the Social Phobia Inventory (SPIN). New self-rating scale. Br J Psychiatry. 2000 Apr;176:379-86. [doi: [10.1192/bjp.176.4.379](https://doi.org/10.1192/bjp.176.4.379)] [Medline: [10827888](https://pubmed.ncbi.nlm.nih.gov/10827888/)].
